# Crystal structure and Hirshfeld surface analysis of 3-oxours-12-ene-27a,28-dioic acid (quafrinoic acid)

**DOI:** 10.1107/S2056989017006077

**Published:** 2017-04-28

**Authors:** Jean Jules Bankeu Kezetas, Stéphanie Dietagoum Madjouka, Rajesh Kumar, Muhammad Shaiq Ali, Bruno Lenta Njakou, Sammer Yousuf

**Affiliations:** a Department of Chemistry, Faculty of Science, The University of Bamenda, PO Box 39, Bambili, Cameroon; b Department of Chemistry, Faculty of Science, University of Yaoundé I, PO Box 812, Yaoundé, Cameroon; c H.E.J. Research Institute of Chemistry, International Center for Chemical and Biological Sciences, University of Karachi, Karachi 75270, Pakistan; d Department of Chemistry, Higher Teacher Training College, University of Yaounde I, PO Box 47 Yaoundé, Cameroon

**Keywords:** crystal structure, penta­cyclic triterpene, quafrinoic acid, *Nauclea pobeguiniia*, Hirshfeld surface analysis

## Abstract

The title compound, C_30_H_44_O_5_, is a penta­cyclic triterpene isolated from the Cameroonian medicinal plant *Nauclea Pobeguinii* and known as quafrinoic acid. The mol­ecule is composed of five fused six-membered rings, four of which adopt a chair conformation and one a half-chair conformation. Intra­molecular C—H⋯O hydrogen-bond inter­actions exist, which generate *S*6 and *S*8 rings. In the crystal, mol­ecules are linked by pairs of O—H⋯O hydrogen bonds, linking *R*_2_^2^(8) rings into chains running parallel to the *a* axis; these chains are further connected into layers parallel to the *ab* plane by C—H⋯O hydrogen bonds. The Hirshfeld surface analysis of the crystal structure indicates that the most important contributions for the crystal packing are from H⋯H (79.4%) and O⋯H (20.4%) inter­actions.

## Abstract 2

The title compound, known as quafrinoic acid, is a penta­cyclic triterpene isolated from *Nauclea Pobeguinii*. The compound is composed of five fused six-membered rings and the mol­ecular conformation is stabilized by intra­molecular C—H⋯O inter­action, forming *S*6 and *S*8 rings.

## Chemical context   

*Nauclea* is a well-known genus of the Rubiaceae family consisting of 35 species of which ten are distributed in tropical Africa, Asia and Australia (Chen & Taylor, 2011 ▸). Several specimens of this genus, including *Nauclea pobeguinii*, are largely used in traditional medicine in Africa. During the last decade, many studies have been carried out on *N. pobeguinii* to explore its medicinal potential and promising results have made it an attractive target for researchers. The 80% ethano­lic stem bark extract of *N. pobeguinii* has been successfully used in clinical trials for the treatment of uncomplicated malaria (Mesia *et al.*, 2012 ▸). The plant is also reported to have cytotoxic, anti-cancer (Kuete *et al.*, 2015 ▸) and anti-diabetic properties (Agnaniet *et al.*, 2016 ▸). The phytochemical investigations of *N. pobeguinii* have led to the isolation of monoterpene indole alkaloids, triterpenes and phenolic compounds (Kuete *et al.*, 2015 ▸; Xu *et al.*, 2012 ▸; Zeches *et al.*, 1985 ▸). In a continuation of our phytochemical investigation of Cameroonian medicinal plants, we have examined the stem bark of *N. pobeguinii* and isolated quafrinoic acid. Although the atomic connectivity of quafrinoic acid has already been determined by spectroscopic methods (Ajaiyeoba & Krebs, 2003 ▸), we report herein the single crystal X-ray diffraction structure and Hirshfeld surface analysis of quafrinoic acid for the first time.
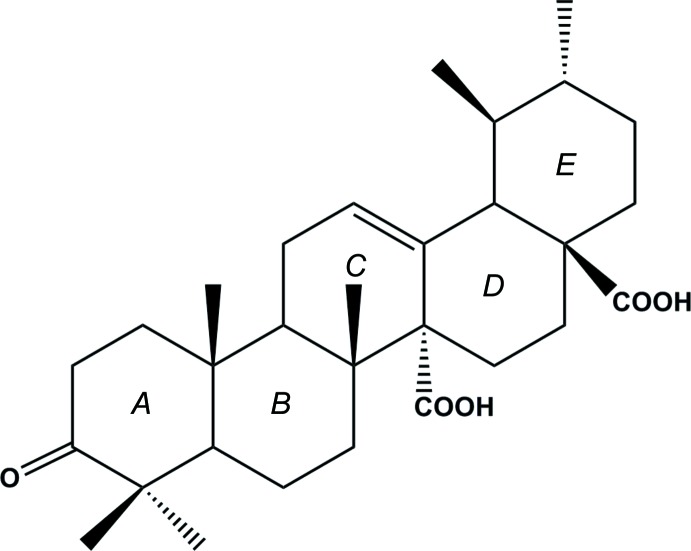


## Structural commentary   

**Figure 1:**
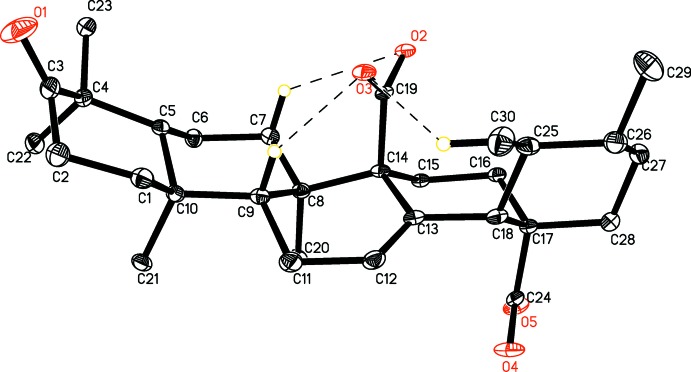
The mol­ecular structure of the title compound, with displacement ellipsoids drawn at the 30% probability level. Dashed lines indicate intra­molecular hydrogen bonds. H atoms not involved in hydrogen bonding have been omitted.

The title compound C_30_H_44_O_5_, is a penta­cyclic triterpene composed of five fused six-membered rings *A* (C1–C5/C10), *B* (C5–C10), *C* (C8–C9/C11–C14), *D* (C14–C18) and *E* (C17–C18/C25–C28) (Fig. 1 ▸). Rings *A*, *B*, *D* and *E* each exhibit a chair conformation, whereas ring *C* has a half-chair conformation. Rings *A*/*B*, *B*/*C* and *C*/*D* are *trans* fused to each other along the C5—C10, C8—C9, and C13—C14 bonds, respectively. Rings *D* and *E* are *cis* fused along the C17—C18 bond along with the axially oriented carb­oxy­lic acid functionalities at C14 and C17. The bond dimensions are similar to those found in structurally related compounds (Csuk *et al.*, 2015 ▸; Awang *et al.*, 2009 ▸).

The mol­ecular conformation is stabilized by intra­molecular hydrogen-bonding inter­actions involving as acceptors the oxygen atoms of the axially oriented carb­oxy­lic group O2/O3/C19 *via* C7—H7*A*⋯O3, C9—H9*A*⋯O3 and C30—H30*A*⋯O3 hydrogen bonds and forming rings with *S*(6), *S*(6) and *S*(8) graph-set motifs, respectively (Table 1 ▸).

## Supra­molecular features   

**Figure 2:**
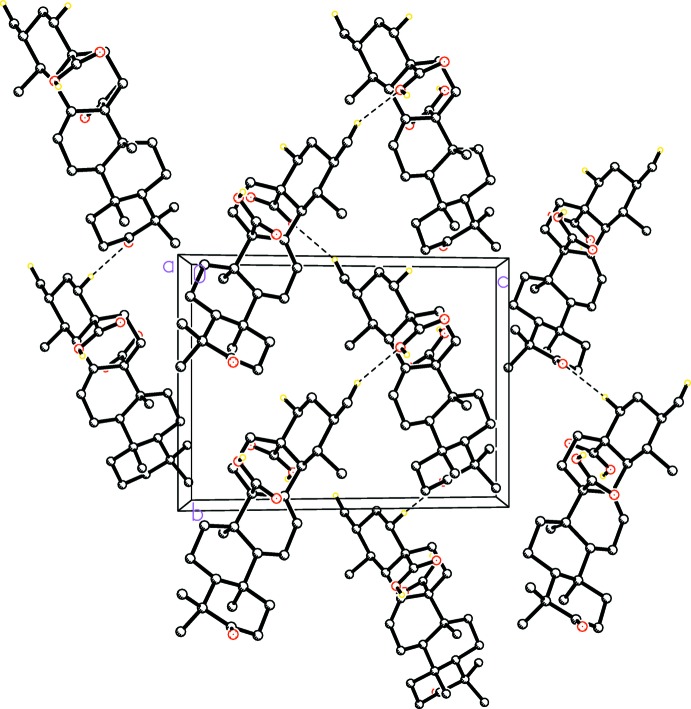
The crystal packing of the title compound viewed down the *a* axis. Only H atoms involved in hydrogen bonding are shown.

In the crystal, mol­ecules are linked into chains parallel to the *a* axis through pairs of O—H⋯O hydrogen bonds, forming 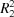
(8) rings. These chains are further connected into layers parallel to the *ab* plane by C—H⋯O hydrogen bonds (Table 1 ▸; Fig. 2 ▸).

## Hirshfeld surface analysis   

**Figure 3:**
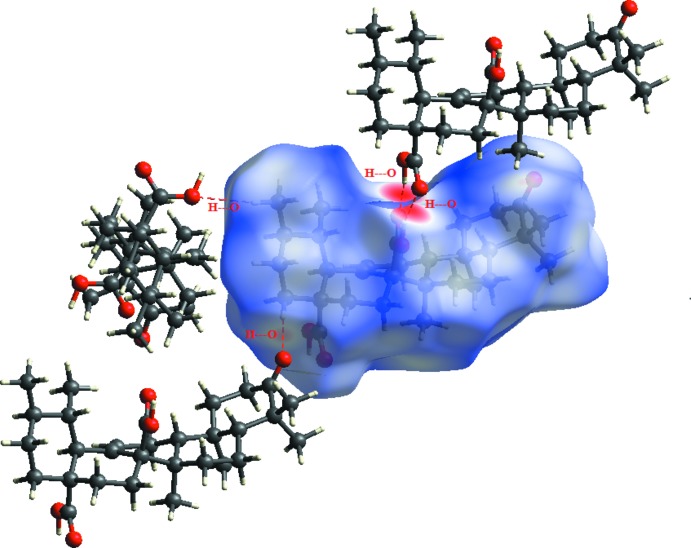
*d*_norm_ mapped on Hirshfeld surface for visualizing the inter-contacts of the title compound. Dashed lines indicate hydrogen bonds.

**Figure 4:**
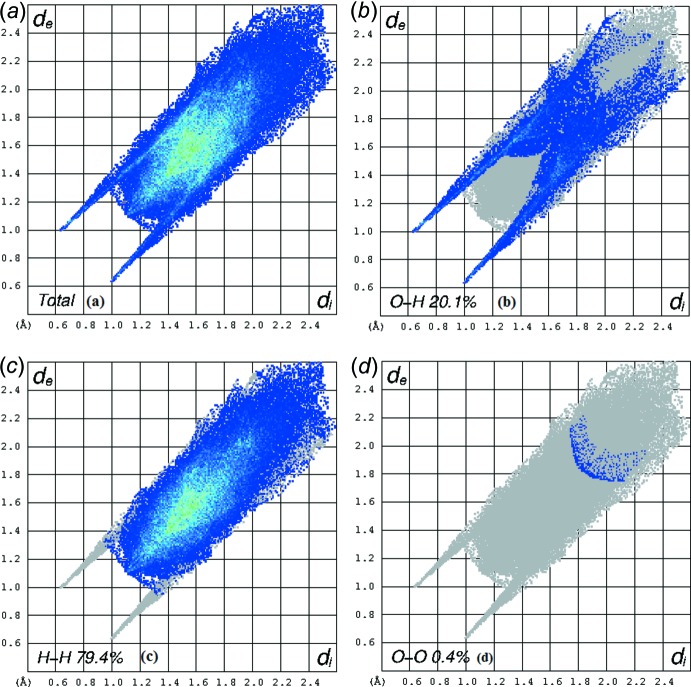
Two-dimensional fingerprint plot analysis of (*a*) all inter­actions, (*b*) H⋯H contacts, (*c*) O⋯H contacts and (*d*) O⋯O contacts. The outline of the full fingerprint plots is shown in grey. *d*_i_ is the closet inter­nal distance from a given point on the Hirshfeld surface and *d*_e_ is the closest external contact.

An Hirshfeld surface analysis (Hirshfeld, 1977 ▸; Spackman & Jayatilaka, 2009 ▸) of the title compound was carried out (Fig. 3 ▸) to investigate the location of atoms with potential to form hydrogen bonds and the qu­anti­tative ratio of these inter­actions. The analysis of the crystal structure suggests that the most important inter­action is H⋯H contributing 79.4% to the overall crystal packing. The other important inter­action is O⋯H, contributing 20.4% towards the crystal packing. The weakest inter­molecular contact for the cohesion of the structure is O⋯O, found to contribute only 0.4%. The graphical representation of the Hirshfeld surface (Fig. 4 ▸) suggests the locations of inter­molecular contacts. These contacts are represented by conventional mapping of *d*_norm_ on mol­ecular Hirshfeld surfaces as shown in Fig. 3 ▸. The H⋯H contribution for the crystal packing is shown as a Hirshfeld surface two-dimensional fingerprint plot with red dots (Wolff *et al.*, 2012 ▸). The *d*_e_ (*y* axis) and *d*_i_ (*x* axis) values are the closest external and inter­nal distances (Å) from a given points on the Hirshfeld surface contacts (Fig. 4 ▸).

## Synthesis and crystallization   

The stem bark of *N. pobeguinii* (Pobég. ex Pellegr.) Merr. ex E·M.A., Rubiaceae, were collected in March 2015 from Makénéné, Centre Region of Cameroon, identified by Dr Njouonkou André Ledoux and Mr Tacham Walter Ndam, lecturers in botany at the Department of Biological Sciences, Faculty of Science, The University of Bamenda, and compared with voucher specimens formerly kept at the National Herbarium under the registration number (32597/HNC). 7.2 kg of the air-dried and ground stem bark of *N. pobeguinii* was extracted with MeOH (3 × 20 L) at room temperature and allowed to concentrate under reduced pressure at low temperature to obtain 1000 g of brown crude extract. The extract was subjected to medium pressure liquid column chromatography over silica gel (Merck, 230–400 mesh) eluting with *n*-hexane, *n*-hexa­ne/EtOAc, EtOAc and EtOAc/MeOH, in increasing order of polarity to yield quafrinoic acid (25 mg). The purified compound was recrystallized by slow evaporation of a methanol solution at room temperature.

## Refinement   

Crystal data, data collection and structure refinement details are summarized in Table 2 ▸. H atoms on methyl, methyl­ene and methine carbon atoms were positioned geometrically with C—H = 0.96 Å (CH_3_), 0.97 Å (CH_2_) and 0.93 Å (CH) and constrained to ride on their parent atoms with *U*_iso_(H)= 1.2*U*_eq_(C) or 1.5*U*_eq_(C) for methyl H atoms. The carb­oxy H atoms were located in a difference-Fourier map and refined isotropically, with the O4—H4 bond length constrained to be 0.90 (1) Å.

## Supplementary Material

Crystal structure: contains datablock(s) global, I. DOI: 10.1107/S2056989017006077/rz5213sup1.cif

Structure factors: contains datablock(s) I. DOI: 10.1107/S2056989017006077/rz5213Isup2.hkl

Supporting information file. DOI: 10.1107/S2056989017006077/rz5213Isup3.cml

CCDC reference: 1545425

Additional supporting information:  crystallographic information; 3D view; checkCIF report

## Figures and Tables

**Table 1:** Hydrogen-bond geometry (Å, °)

*D*—H⋯*A*	*D*—H	H⋯*A*	*D*⋯*A*	*D*—H⋯*A*
O4—H4*A*⋯O3^i^	0.91 (1)	1.70 (1)	2.6046 (18)	172 (5)
O2—H19*A*⋯O5^ii^	0.80 (4)	1.89 (4)	2.6702 (18)	165 (4)
C7—H7*A*⋯O2	0.99	2.54	3.232 (2)	127
C9—H9*A*⋯O3	1.00	2.18	3.009 (2)	139
C28—H28*B*⋯O1^iii^	0.99	2.49	3.477 (3)	173
C29—H29*A*⋯O4^iv^	0.98	2.57	3.497 (3)	158
C30—H30*A*⋯O3	0.98	2.58	3.221 (3)	123

Symmetry codes: (i) 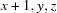; (ii) 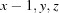; (iii) 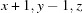; (iv) 
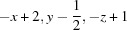
.

**Table 2:** Experimental details

Crystal data	
Chemical formula	C_30_H_44_O_5_
*M*_r_	484.65
Crystal system, space group	Monoclinic, *P*2_1_
Temperature (K)	100
*a*, *b*, *c* (Å)	8.3465 (2), 10.9783 (3), 14.6583 (4)
β (°)	101.056 (1)
*V* (Å^3^)	1318.22 (6)
*Z*	2
Radiation type	Cu *K*α
μ (mm^−1^)	0.64
Crystal size (mm)	0.45 × 0.23 × 0.12

Data collection	
Diffractometer	Bruker SMART APEX CCD area-detector
Absorption correction	Multi-scan (*SADABS*; Bruker, 2009 ▸)
*T*_min_, *T*_max_	0.760, 0.927
No. of measured, independent and observed [*I* > 2σ(*I*)] reflections	28696, 5116, 5023
*R*_int_	0.042
(sin θ/λ)_max_ (Å^−1^)	0.618

Refinement	
*R*[*F* ^2^ > 2σ(*F* ^2^)], *wR*(*F* ^2^), *S*	0.041, 0.115, 1.06
No. of reflections	5116
No. of parameters	325
No. of restraints	2
H-atom treatment	H atoms treated by a mixture of independent and constrained refinement
Δρ_max_, Δρ_min_ (e Å^−3^)	0.70, −0.31
Absolute structure	Flack, 1983 ▸
Absolute structure parameter	0.14 (17)

Computer programs: *SMART* and *SAINT* (Bruker, 2009 ▸), *SHELXTL* (Sheldrick, 2008 ▸), *PLATON* (Spek, 2009 ▸) and *publCIF* (Westrip, 2010 ▸).

## supplementary crystallographic information

### Crystal data

**Table:**

C_30_H_44_O_5_	*F*(000) = 528
*M**_r_* = 484.65	*D*_x_ = 1.221 Mg m^−^^3^
Monoclinic, *P*2_1_	Cu *K*α radiation, λ = 1.54178 Å
*a* = 8.3465 (2) Å	Cell parameters from 9925 reflections
*b* = 10.9783 (3) Å	θ = 5.1–72.1°
*c* = 14.6583 (4) Å	µ = 0.64 mm^−^^1^
β = 101.056 (1)°	*T* = 100 K
*V* = 1318.22 (6) Å^3^	Block, colourless
*Z* = 2	0.45 × 0.23 × 0.12 mm

### Data collection

**Table:**

Bruker SMART APEX CCD area-detector diffractometer	5116 independent reflections
Radiation source: fine-focus sealed tube	5023 reflections with *I* > 2σ(*I*)
Graphite monochromator	*R*_int_ = 0.042
ω scans	θ_max_ = 72.2°, θ_min_ = 3.1°
Absorption correction: multi-scan (SADABS; Bruker, 2009)	*h* = −10→10
*T*_min_ = 0.760, *T*_max_ = 0.927	*k* = −12→13
28696 measured reflections	*l* = −18→18

### Refinement

**Table:**

Refinement on *F*^2^	Hydrogen site location: inferred from neighbouring sites
Least-squares matrix: full	H atoms treated by a mixture of independent and constrained refinement
*R*[*F*^2^ > 2σ(*F*^2^)] = 0.041	*w* = 1/[σ^2^(*F*_o_^2^) + (0.0718*P*)^2^ + 0.4209*P*] where *P* = (*F*_o_^2^ + 2*F*_c_^2^)/3
*wR*(*F*^2^) = 0.115	(Δ/σ)_max_ < 0.001
*S* = 1.06	Δρ_max_ = 0.70 e Å^−^^3^
5116 reflections	Δρ_min_ = −0.31 e Å^−^^3^
325 parameters	Extinction correction: SHELXTL (Sheldrick, 2008), Fc^*^=kFc[1+0.001xFc^2^λ^3^/sin(2θ)]^-1/4^
2 restraints	Extinction coefficient: 0.0047 (12)
Primary atom site location: structure-invariant direct methods	Absolute structure: Flack, 1983
Secondary atom site location: difference Fourier map	Absolute structure parameter: 0.14 (17)

### Special details

**Table:**

Geometry. All esds (except the esd in the dihedral angle between two l.s. planes) are estimated using the full covariance matrix. The cell esds are taken into account individually in the estimation of esds in distances, angles and torsion angles; correlations between esds in cell parameters are only used when they are defined by crystal symmetry. An approximate (isotropic) treatment of cell esds is used for estimating esds involving l.s. planes.
Refinement. Refinement of F^2^ against ALL reflections. The weighted R-factor wR and goodness of fit S are based on F^2^, conventional R-factors R are based on F, with F set to zero for negative F^2^. The threshold expression of F^2^ > 2sigma(F^2^) is used only for calculating R-factors(gt) etc. and is not relevant to the choice of reflections for refinement. R-factors based on F^2^ are statistically about twice as large as those based on F, and R- factors based on ALL data will be even larger.

### Fractional atomic coordinates and isotropic or equivalent isotropic displacement parameters (Å^2^)

**Table:**

	*x*	*y*	*z*	*U*_iso_*/*U*_eq_	
O1	0.27554 (19)	0.9227 (2)	0.81577 (14)	0.0587 (6)	
O2	0.63334 (15)	0.29733 (12)	0.81088 (9)	0.0213 (3)	
O3	0.56617 (14)	0.43709 (13)	0.70046 (8)	0.0232 (3)	
O4	1.26493 (17)	0.36149 (16)	0.66220 (11)	0.0376 (4)	
O5	1.31424 (15)	0.25278 (16)	0.79245 (10)	0.0328 (3)	
C1	0.6083 (2)	0.84362 (18)	0.72071 (13)	0.0250 (4)	
H1A	0.6715	0.8743	0.6748	0.030*	
H1B	0.5291	0.7829	0.6889	0.030*	
C2	0.5145 (2)	0.9494 (2)	0.75258 (15)	0.0315 (4)	
H2A	0.5919	1.0146	0.7786	0.038*	
H2B	0.4378	0.9833	0.6986	0.038*	
C3	0.4206 (2)	0.90781 (18)	0.82544 (14)	0.0295 (4)	
C4	0.5182 (2)	0.84228 (17)	0.91042 (13)	0.0235 (4)	
C5	0.62587 (19)	0.74263 (16)	0.87525 (12)	0.0190 (3)	
H5A	0.5453	0.6818	0.8430	0.023*	
C6	0.7330 (2)	0.66974 (17)	0.95315 (12)	0.0217 (4)	
H6A	0.8352	0.7148	0.9770	0.026*	
H6B	0.6745	0.6572	1.0051	0.026*	
C7	0.7722 (2)	0.54683 (17)	0.91361 (11)	0.0206 (3)	
H7A	0.6692	0.5010	0.8938	0.025*	
H7B	0.8419	0.4994	0.9634	0.025*	
C8	0.85976 (19)	0.55753 (17)	0.83033 (11)	0.0186 (3)	
C9	0.7771 (2)	0.65618 (17)	0.75961 (11)	0.0192 (3)	
H9A	0.6722	0.6185	0.7277	0.023*	
C10	0.7266 (2)	0.77963 (16)	0.80027 (12)	0.0204 (4)	
C11	0.8786 (2)	0.67108 (18)	0.68322 (14)	0.0281 (4)	
H11A	0.8169	0.7212	0.6321	0.034*	
H11B	0.9813	0.7144	0.7090	0.034*	
C12	0.9178 (2)	0.55000 (19)	0.64540 (13)	0.0262 (4)	
H12A	0.9584	0.5508	0.5891	0.031*	
C13	0.90136 (19)	0.44199 (17)	0.68303 (11)	0.0195 (3)	
C14	0.84932 (18)	0.42886 (17)	0.77766 (11)	0.0175 (3)	
C15	0.96004 (19)	0.33334 (16)	0.83675 (11)	0.0171 (3)	
H15A	1.0692	0.3697	0.8584	0.021*	
H15B	0.9137	0.3141	0.8924	0.021*	
C16	0.98026 (19)	0.21453 (16)	0.78546 (12)	0.0185 (3)	
H16A	0.8734	0.1728	0.7695	0.022*	
H16B	1.0563	0.1601	0.8269	0.022*	
C17	1.0464 (2)	0.23782 (16)	0.69610 (12)	0.0194 (4)	
C18	0.9318 (2)	0.32589 (17)	0.63186 (12)	0.0211 (4)	
H18A	0.9905	0.3507	0.5814	0.025*	
C19	0.6708 (2)	0.38454 (17)	0.76152 (11)	0.0174 (3)	
C20	1.0395 (2)	0.59073 (18)	0.86730 (14)	0.0258 (4)	
H20A	1.0453	0.6693	0.8996	0.039*	
H20B	1.0973	0.5964	0.8152	0.039*	
H20C	1.0904	0.5277	0.9107	0.039*	
C21	0.8701 (2)	0.86653 (17)	0.83604 (14)	0.0254 (4)	
H21A	0.9272	0.8856	0.7853	0.038*	
H21B	0.9460	0.8273	0.8869	0.038*	
H21C	0.8283	0.9419	0.8587	0.038*	
C22	0.6152 (2)	0.93770 (19)	0.97522 (14)	0.0301 (4)	
H22A	0.5396	0.9967	0.9940	0.045*	
H22B	0.6908	0.9801	0.9425	0.045*	
H22C	0.6770	0.8972	1.0305	0.045*	
C23	0.3981 (2)	0.78061 (19)	0.96218 (14)	0.0283 (4)	
H23A	0.3303	0.8426	0.9844	0.042*	
H23B	0.4587	0.7351	1.0153	0.042*	
H23C	0.3284	0.7246	0.9201	0.042*	
C24	1.2189 (2)	0.29000 (17)	0.71987 (12)	0.0206 (3)	
C25	0.7705 (2)	0.26347 (19)	0.58354 (13)	0.0248 (4)	
H25A	0.7031	0.2461	0.6316	0.030*	
C26	0.8008 (3)	0.1421 (2)	0.53560 (14)	0.0326 (5)	
H26A	0.8574	0.1613	0.4831	0.039*	
C27	0.9097 (2)	0.0580 (2)	0.60201 (14)	0.0307 (4)	
H27A	0.9316	−0.0164	0.5682	0.037*	
H27B	0.8519	0.0331	0.6520	0.037*	
C28	1.0706 (2)	0.11713 (18)	0.64521 (14)	0.0266 (4)	
H28A	1.1338	0.1340	0.5959	0.032*	
H28B	1.1348	0.0598	0.6900	0.032*	
C29	0.6414 (3)	0.0764 (3)	0.49574 (18)	0.0475 (6)	
H29A	0.6656	0.0004	0.4659	0.071*	
H29B	0.5734	0.1287	0.4497	0.071*	
H29C	0.5830	0.0578	0.5460	0.071*	
C30	0.6761 (3)	0.3486 (2)	0.51214 (15)	0.0378 (5)	
H30A	0.6568	0.4257	0.5420	0.057*	
H30B	0.5712	0.3114	0.4847	0.057*	
H30C	0.7389	0.3642	0.4633	0.057*	
H19A	0.539 (5)	0.281 (4)	0.796 (3)	0.082 (12)*	
H4A	1.367 (3)	0.391 (5)	0.680 (4)	0.14 (2)*	

### Atomic displacement parameters (Å^2^)

**Table:**

	*U*^11^	*U*^22^	*U*^33^	*U*^12^	*U*^13^	*U*^23^
O1	0.0238 (8)	0.0760 (14)	0.0769 (12)	0.0147 (8)	0.0113 (8)	0.0335 (11)
O2	0.0138 (5)	0.0251 (7)	0.0263 (6)	−0.0035 (5)	0.0067 (4)	0.0021 (5)
O3	0.0123 (5)	0.0291 (7)	0.0266 (6)	−0.0014 (5)	−0.0003 (4)	0.0041 (5)
O4	0.0185 (6)	0.0464 (10)	0.0495 (9)	−0.0092 (6)	0.0106 (6)	0.0124 (7)
O5	0.0139 (6)	0.0527 (9)	0.0318 (7)	−0.0003 (6)	0.0041 (5)	0.0018 (6)
C1	0.0204 (8)	0.0245 (9)	0.0283 (9)	−0.0002 (7)	0.0004 (7)	0.0056 (7)
C2	0.0255 (9)	0.0282 (11)	0.0386 (10)	0.0046 (8)	0.0002 (8)	0.0076 (8)
C3	0.0225 (9)	0.0230 (10)	0.0414 (10)	0.0048 (7)	0.0024 (8)	0.0016 (8)
C4	0.0178 (8)	0.0217 (9)	0.0303 (9)	0.0001 (7)	0.0030 (7)	−0.0035 (7)
C5	0.0146 (7)	0.0187 (8)	0.0229 (8)	−0.0023 (6)	0.0019 (6)	−0.0014 (6)
C6	0.0226 (8)	0.0214 (9)	0.0206 (8)	−0.0003 (7)	0.0029 (7)	−0.0018 (7)
C7	0.0209 (8)	0.0211 (9)	0.0191 (7)	−0.0016 (7)	0.0026 (6)	0.0017 (7)
C8	0.0143 (7)	0.0198 (8)	0.0212 (8)	−0.0002 (6)	0.0020 (6)	0.0025 (7)
C9	0.0162 (7)	0.0204 (9)	0.0210 (8)	−0.0015 (6)	0.0034 (6)	0.0041 (7)
C10	0.0163 (7)	0.0171 (9)	0.0273 (8)	−0.0006 (6)	0.0026 (6)	0.0029 (7)
C11	0.0308 (9)	0.0240 (10)	0.0328 (10)	0.0003 (8)	0.0141 (8)	0.0095 (7)
C12	0.0259 (9)	0.0295 (10)	0.0265 (9)	0.0002 (8)	0.0137 (7)	0.0041 (8)
C13	0.0124 (7)	0.0265 (9)	0.0199 (8)	−0.0019 (7)	0.0042 (6)	0.0025 (7)
C14	0.0103 (7)	0.0217 (8)	0.0202 (7)	−0.0022 (6)	0.0021 (5)	0.0028 (7)
C15	0.0118 (7)	0.0209 (8)	0.0186 (7)	−0.0014 (6)	0.0028 (6)	0.0021 (6)
C16	0.0134 (7)	0.0200 (9)	0.0226 (8)	−0.0007 (6)	0.0043 (6)	0.0025 (7)
C17	0.0136 (7)	0.0224 (9)	0.0231 (8)	−0.0024 (6)	0.0057 (6)	−0.0021 (7)
C18	0.0157 (7)	0.0294 (10)	0.0192 (8)	−0.0032 (7)	0.0058 (6)	0.0013 (7)
C19	0.0123 (7)	0.0225 (9)	0.0186 (7)	−0.0009 (6)	0.0059 (6)	0.0000 (6)
C20	0.0160 (8)	0.0222 (9)	0.0369 (10)	−0.0025 (6)	−0.0006 (7)	−0.0020 (7)
C21	0.0189 (8)	0.0196 (9)	0.0371 (10)	−0.0039 (7)	0.0040 (7)	0.0023 (7)
C22	0.0266 (9)	0.0245 (10)	0.0382 (10)	−0.0022 (8)	0.0036 (8)	−0.0081 (8)
C23	0.0249 (9)	0.0262 (10)	0.0355 (10)	−0.0009 (8)	0.0101 (7)	−0.0057 (8)
C24	0.0156 (7)	0.0243 (9)	0.0237 (8)	−0.0002 (7)	0.0082 (6)	−0.0025 (7)
C25	0.0173 (8)	0.0331 (10)	0.0228 (8)	−0.0050 (7)	0.0010 (6)	−0.0005 (7)
C26	0.0323 (10)	0.0416 (12)	0.0255 (9)	−0.0099 (9)	0.0094 (8)	−0.0086 (9)
C27	0.0333 (10)	0.0301 (11)	0.0316 (10)	−0.0093 (9)	0.0137 (8)	−0.0097 (8)
C28	0.0243 (9)	0.0261 (10)	0.0317 (10)	−0.0007 (7)	0.0109 (7)	−0.0041 (8)
C29	0.0463 (13)	0.0494 (15)	0.0411 (12)	−0.0124 (12)	−0.0062 (10)	−0.0105 (11)
C30	0.0314 (10)	0.0439 (13)	0.0355 (11)	0.0079 (9)	0.0003 (8)	−0.0029 (9)

### Geometric parameters (Å, º)

**Table:**

O1—C3	1.203 (3)	C14—C15	1.548 (2)
O2—C19	1.275 (2)	C15—C16	1.531 (2)
O2—H19A	0.79 (4)	C15—H15A	0.9900
O3—C19	1.264 (2)	C15—H15B	0.9900
O4—C24	1.266 (2)	C16—C17	1.537 (2)
O4—H4A	0.906 (10)	C16—H16A	0.9900
O5—C24	1.268 (2)	C16—H16B	0.9900
C1—C2	1.523 (3)	C17—C24	1.526 (2)
C1—C10	1.545 (2)	C17—C18	1.546 (2)
C1—H1A	0.9900	C17—C28	1.553 (3)
C1—H1B	0.9900	C18—C25	1.555 (2)
C2—C3	1.511 (3)	C18—H18A	1.0000
C2—H2A	0.9900	C20—H20A	0.9800
C2—H2B	0.9900	C20—H20B	0.9800
C3—C4	1.531 (3)	C20—H20C	0.9800
C4—C23	1.526 (3)	C21—H21A	0.9800
C4—C22	1.536 (3)	C21—H21B	0.9800
C4—C5	1.565 (2)	C21—H21C	0.9800
C5—C6	1.534 (2)	C22—H22A	0.9800
C5—C10	1.560 (2)	C22—H22B	0.9800
C5—H5A	1.0000	C22—H22C	0.9800
C6—C7	1.529 (2)	C23—H23A	0.9800
C6—H6A	0.9900	C23—H23B	0.9800
C6—H6B	0.9900	C23—H23C	0.9800
C7—C8	1.543 (2)	C25—C30	1.508 (3)
C7—H7A	0.9900	C25—C26	1.550 (3)
C7—H7B	0.9900	C25—H25A	1.0000
C8—C20	1.538 (2)	C26—C27	1.512 (3)
C8—C9	1.565 (2)	C26—C29	1.528 (3)
C8—C14	1.604 (2)	C26—H26A	1.0000
C9—C11	1.537 (2)	C27—C28	1.517 (3)
C9—C10	1.571 (2)	C27—H27A	0.9900
C9—H9A	1.0000	C27—H27B	0.9900
C10—C21	1.543 (2)	C28—H28A	0.9900
C11—C12	1.501 (3)	C28—H28B	0.9900
C11—H11A	0.9900	C29—H29A	0.9800
C11—H11B	0.9900	C29—H29B	0.9800
C12—C13	1.326 (3)	C29—H29C	0.9800
C12—H12A	0.9500	C30—H30A	0.9800
C13—C18	1.525 (3)	C30—H30B	0.9800
C13—C14	1.538 (2)	C30—H30C	0.9800
C14—C19	1.542 (2)		
			
C19—O2—H19A	111 (3)	C15—C16—H16A	109.3
C24—O4—H4A	115 (3)	C17—C16—H16A	109.3
C2—C1—C10	113.99 (16)	C15—C16—H16B	109.3
C2—C1—H1A	108.8	C17—C16—H16B	109.3
C10—C1—H1A	108.8	H16A—C16—H16B	108.0
C2—C1—H1B	108.8	C24—C17—C16	110.18 (13)
C10—C1—H1B	108.8	C24—C17—C18	110.52 (14)
H1A—C1—H1B	107.6	C16—C17—C18	109.97 (13)
C3—C2—C1	110.69 (17)	C24—C17—C28	103.06 (13)
C3—C2—H2A	109.5	C16—C17—C28	111.63 (15)
C1—C2—H2A	109.5	C18—C17—C28	111.31 (14)
C3—C2—H2B	109.5	C13—C18—C17	111.42 (13)
C1—C2—H2B	109.5	C13—C18—C25	112.39 (14)
H2A—C2—H2B	108.1	C17—C18—C25	112.48 (15)
O1—C3—C2	121.49 (18)	C13—C18—H18A	106.7
O1—C3—C4	121.75 (19)	C17—C18—H18A	106.7
C2—C3—C4	116.73 (16)	C25—C18—H18A	106.7
C23—C4—C3	108.39 (15)	O3—C19—O2	122.26 (15)
C23—C4—C22	108.26 (15)	O3—C19—C14	118.73 (15)
C3—C4—C22	108.53 (16)	O2—C19—C14	119.00 (15)
C23—C4—C5	109.09 (15)	C8—C20—H20A	109.5
C3—C4—C5	108.06 (15)	C8—C20—H20B	109.5
C22—C4—C5	114.37 (14)	H20A—C20—H20B	109.5
C6—C5—C10	110.16 (13)	C8—C20—H20C	109.5
C6—C5—C4	114.15 (14)	H20A—C20—H20C	109.5
C10—C5—C4	118.11 (14)	H20B—C20—H20C	109.5
C6—C5—H5A	104.2	C10—C21—H21A	109.5
C10—C5—H5A	104.2	C10—C21—H21B	109.5
C4—C5—H5A	104.2	H21A—C21—H21B	109.5
C7—C6—C5	108.36 (13)	C10—C21—H21C	109.5
C7—C6—H6A	110.0	H21A—C21—H21C	109.5
C5—C6—H6A	110.0	H21B—C21—H21C	109.5
C7—C6—H6B	110.0	C4—C22—H22A	109.5
C5—C6—H6B	110.0	C4—C22—H22B	109.5
H6A—C6—H6B	108.4	H22A—C22—H22B	109.5
C6—C7—C8	113.65 (15)	C4—C22—H22C	109.5
C6—C7—H7A	108.8	H22A—C22—H22C	109.5
C8—C7—H7A	108.8	H22B—C22—H22C	109.5
C6—C7—H7B	108.8	C4—C23—H23A	109.5
C8—C7—H7B	108.8	C4—C23—H23B	109.5
H7A—C7—H7B	107.7	H23A—C23—H23B	109.5
C20—C8—C7	108.50 (14)	C4—C23—H23C	109.5
C20—C8—C9	110.22 (14)	H23A—C23—H23C	109.5
C7—C8—C9	111.18 (13)	H23B—C23—H23C	109.5
C20—C8—C14	109.73 (14)	O4—C24—O5	122.53 (16)
C7—C8—C14	108.88 (14)	O4—C24—C17	118.30 (15)
C9—C8—C14	108.31 (12)	O5—C24—C17	118.91 (15)
C11—C9—C8	108.79 (14)	C30—C25—C26	109.11 (16)
C11—C9—C10	114.23 (15)	C30—C25—C18	109.54 (17)
C8—C9—C10	117.53 (13)	C26—C25—C18	112.50 (15)
C11—C9—H9A	105.0	C30—C25—H25A	108.5
C8—C9—H9A	105.0	C26—C25—H25A	108.5
C10—C9—H9A	105.0	C18—C25—H25A	108.5
C21—C10—C1	108.51 (14)	C27—C26—C29	109.2 (2)
C21—C10—C5	114.20 (14)	C27—C26—C25	111.32 (15)
C1—C10—C5	107.32 (13)	C29—C26—C25	111.93 (19)
C21—C10—C9	114.51 (14)	C27—C26—H26A	108.1
C1—C10—C9	106.57 (14)	C29—C26—H26A	108.1
C5—C10—C9	105.25 (13)	C25—C26—H26A	108.1
C12—C11—C9	111.40 (15)	C26—C27—C28	112.44 (17)
C12—C11—H11A	109.3	C26—C27—H27A	109.1
C9—C11—H11A	109.3	C28—C27—H27A	109.1
C12—C11—H11B	109.3	C26—C27—H27B	109.1
C9—C11—H11B	109.3	C28—C27—H27B	109.1
H11A—C11—H11B	108.0	H27A—C27—H27B	107.8
C13—C12—C11	126.23 (15)	C27—C28—C17	112.29 (15)
C13—C12—H12A	116.9	C27—C28—H28A	109.1
C11—C12—H12A	116.9	C17—C28—H28A	109.1
C12—C13—C18	120.17 (15)	C27—C28—H28B	109.1
C12—C13—C14	121.93 (17)	C17—C28—H28B	109.1
C18—C13—C14	117.89 (15)	H28A—C28—H28B	107.9
C13—C14—C19	108.85 (13)	C26—C29—H29A	109.5
C13—C14—C15	109.06 (13)	C26—C29—H29B	109.5
C19—C14—C15	109.13 (14)	H29A—C29—H29B	109.5
C13—C14—C8	110.66 (14)	C26—C29—H29C	109.5
C19—C14—C8	108.26 (13)	H29A—C29—H29C	109.5
C15—C14—C8	110.83 (12)	H29B—C29—H29C	109.5
C16—C15—C14	114.36 (13)	C25—C30—H30A	109.5
C16—C15—H15A	108.7	C25—C30—H30B	109.5
C14—C15—H15A	108.7	H30A—C30—H30B	109.5
C16—C15—H15B	108.7	C25—C30—H30C	109.5
C14—C15—H15B	108.7	H30A—C30—H30C	109.5
H15A—C15—H15B	107.6	H30B—C30—H30C	109.5
C15—C16—C17	111.60 (14)		
			
C10—C1—C2—C3	−56.5 (2)	C20—C8—C14—C13	−73.60 (17)
C1—C2—C3—O1	−124.2 (2)	C7—C8—C14—C13	167.79 (13)
C1—C2—C3—C4	53.9 (2)	C9—C8—C14—C13	46.77 (16)
O1—C3—C4—C23	12.1 (3)	C20—C8—C14—C19	167.19 (14)
C2—C3—C4—C23	−165.95 (17)	C7—C8—C14—C19	48.58 (16)
O1—C3—C4—C22	−105.2 (2)	C9—C8—C14—C19	−72.45 (15)
C2—C3—C4—C22	76.7 (2)	C20—C8—C14—C15	47.54 (17)
O1—C3—C4—C5	130.2 (2)	C7—C8—C14—C15	−71.08 (15)
C2—C3—C4—C5	−47.9 (2)	C9—C8—C14—C15	167.90 (12)
C23—C4—C5—C6	−63.04 (19)	C13—C14—C15—C16	−48.13 (18)
C3—C4—C5—C6	179.32 (15)	C19—C14—C15—C16	70.66 (16)
C22—C4—C5—C6	58.3 (2)	C8—C14—C15—C16	−170.20 (12)
C23—C4—C5—C10	165.07 (15)	C14—C15—C16—C17	56.47 (17)
C3—C4—C5—C10	47.43 (19)	C15—C16—C17—C24	64.84 (18)
C22—C4—C5—C10	−73.5 (2)	C15—C16—C17—C18	−57.23 (17)
C10—C5—C6—C7	−68.31 (17)	C15—C16—C17—C28	178.72 (14)
C4—C5—C6—C7	156.07 (14)	C12—C13—C18—C17	130.96 (17)
C5—C6—C7—C8	58.19 (18)	C14—C13—C18—C17	−49.90 (18)
C6—C7—C8—C20	76.36 (18)	C12—C13—C18—C25	−101.75 (19)
C6—C7—C8—C9	−45.00 (19)	C14—C13—C18—C25	77.39 (18)
C6—C7—C8—C14	−164.25 (13)	C24—C17—C18—C13	−68.80 (17)
C20—C8—C9—C11	54.48 (19)	C16—C17—C18—C13	53.07 (18)
C7—C8—C9—C11	174.83 (15)	C28—C17—C18—C13	177.31 (13)
C14—C8—C9—C11	−65.58 (17)	C24—C17—C18—C25	163.96 (13)
C20—C8—C9—C10	−77.29 (18)	C16—C17—C18—C25	−74.17 (17)
C7—C8—C9—C10	43.06 (19)	C28—C17—C18—C25	50.07 (18)
C14—C8—C9—C10	162.65 (13)	C13—C14—C19—O3	−47.5 (2)
C2—C1—C10—C21	−70.20 (19)	C15—C14—C19—O3	−166.42 (15)
C2—C1—C10—C5	53.7 (2)	C8—C14—C19—O3	72.86 (18)
C2—C1—C10—C9	166.01 (15)	C13—C14—C19—O2	133.37 (16)
C6—C5—C10—C21	−63.99 (19)	C15—C14—C19—O2	14.4 (2)
C4—C5—C10—C21	69.65 (19)	C8—C14—C19—O2	−106.28 (17)
C6—C5—C10—C1	175.69 (14)	C16—C17—C24—O4	−149.64 (17)
C4—C5—C10—C1	−50.67 (19)	C18—C17—C24—O4	−27.9 (2)
C6—C5—C10—C9	62.44 (16)	C28—C17—C24—O4	91.1 (2)
C4—C5—C10—C9	−163.91 (14)	C16—C17—C24—O5	36.0 (2)
C11—C9—C10—C21	−53.9 (2)	C18—C17—C24—O5	157.70 (16)
C8—C9—C10—C21	75.37 (18)	C28—C17—C24—O5	−83.28 (19)
C11—C9—C10—C1	66.10 (18)	C13—C18—C25—C30	61.30 (19)
C8—C9—C10—C1	−164.65 (14)	C17—C18—C25—C30	−171.98 (15)
C11—C9—C10—C5	179.87 (14)	C13—C18—C25—C26	−177.17 (14)
C8—C9—C10—C5	−50.88 (17)	C17—C18—C25—C26	−50.45 (19)
C8—C9—C11—C12	48.4 (2)	C30—C25—C26—C27	174.28 (17)
C10—C9—C11—C12	−178.13 (15)	C18—C25—C26—C27	52.5 (2)
C9—C11—C12—C13	−13.7 (3)	C30—C25—C26—C29	−63.2 (2)
C11—C12—C13—C18	174.45 (18)	C18—C25—C26—C29	175.04 (18)
C11—C12—C13—C14	−4.7 (3)	C29—C26—C27—C28	−179.62 (17)
C12—C13—C14—C19	106.02 (19)	C25—C26—C27—C28	−55.5 (2)
C18—C13—C14—C19	−73.11 (18)	C26—C27—C28—C17	56.3 (2)
C12—C13—C14—C15	−135.01 (17)	C24—C17—C28—C27	−171.43 (15)
C18—C13—C14—C15	45.86 (18)	C16—C17—C28—C27	70.3 (2)
C12—C13—C14—C8	−12.8 (2)	C18—C17—C28—C27	−53.0 (2)
C18—C13—C14—C8	168.04 (13)		

### Hydrogen-bond geometry (Å, º)

**Table:**

*D*—H···*A*	*D*—H	H···*A*	*D*···*A*	*D*—H···*A*
O4—H4*A*···O3^i^	0.91 (1)	1.70 (1)	2.6046 (18)	172 (5)
O2—H19*A*···O5^ii^	0.80 (4)	1.89 (4)	2.6702 (18)	165 (4)
C7—H7*A*···O2	0.99	2.54	3.232 (2)	127
C9—H9*A*···O3	1.00	2.18	3.009 (2)	139
C28—H28*B*···O1^iii^	0.99	2.49	3.477 (3)	173
C29—H29*A*···O4^iv^	0.98	2.57	3.497 (3)	158
C30—H30*A*···O3	0.98	2.58	3.221 (3)	123

Symmetry codes: (i) *x*+1, *y*, *z*; (ii) *x*−1, *y*, *z*; (iii) *x*+1, *y*−1, *z*; (iv) −*x*+2, *y*−1/2, −*z*+1.
